# Editorial: Agri-food innovations in the quest for food and environmental security

**DOI:** 10.3389/fnut.2023.1304246

**Published:** 2023-10-27

**Authors:** Monika Thakur, Miguel A. Prieto, Hao Huang

**Affiliations:** ^1^Amity Institute of Food Technology, Amity University Uttar Pradesh, Noida, India; ^2^Nutrition and Bromatology Group, Department of Analytical Chemistry and Food Science, Faculty of Science, Universidade de Vigo, Ourense, Spain; ^3^College of Ecology, Lishui University, Lishui, China

**Keywords:** food security, food processing, food and health, green processing, food waste, waste valorization, sensors for food

Agri-food systems (AFS) encompass the entirety of activities and components that develop the inter-connectedness in agriculture and food value chain. This comprehensive system encompasses a variety of elements, including technological research and development, input and production processes, agricultural product storage and distribution, processing and packaging, and the various stages of retail, wholesale, catering, and consumption. Moreover, it encompasses critical natural resources like water and soil essential for agricultural and food production, as well as the policies, mechanisms, and cultural traditions associated with agriculture and food. Within this framework, farmers, and stakeholders involved in these related activities, including agricultural institutions and organizations, play a significant role. Additionally, the outcomes of these agricultural and food system activities extend to socio-economic, nutritional, health, and environmental aspects. The Agri-food system's vast scope is pivotal in ensuring food security, enhancing nutritional wellbeing, and fostering human prosperity (1). It not only supplies the essential calories and nutrients required by people but also serves as the foundation of livelihood for small-scale farmers, agricultural small and medium-sized enterprises, and small-scale catering practitioners, among other groups. In essence, the AFS constitutes a comprehensive, multi-agent system with nutritional environment security (Figure 1).

**Figure 1:**
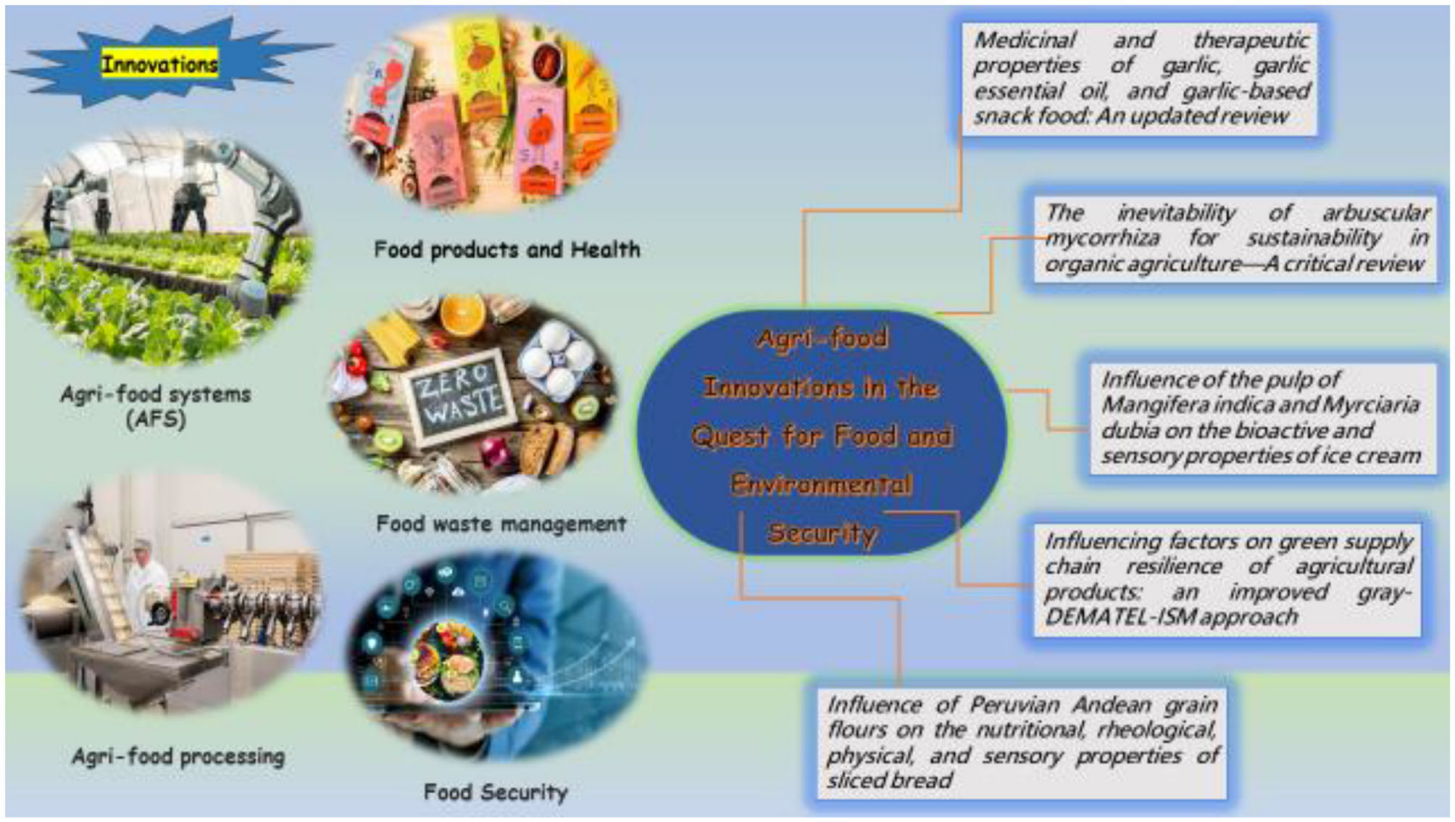
AFS with different parameters in the pursuance of food and environment security.

“*Food security exists when all people, at all times, have physical and economic access to sufficient, safe and nutritious food that meets their dietary needs and food preferences for an active and healthy life*” (2). The AFS of today confront intricate and unprecedented challenges stemming from an array of issues, including climate change, biodiversity loss, migration, conflicts, economic fluctuations, food crises, rapid population growth, biofuel production, suboptimal agricultural practices, food loss, food waste, and the dual pressures of climate change and the COVID-19 pandemic. Simultaneously, global income inequality continues to escalate, with many rural populations living in conditions of poverty or extreme destitution. In the present scenario, our depleted natural resources are no longer sufficient to meet our practical requirements, underscoring the urgent necessity for a profound transformation of the AFS to chart a course toward sustainable, efficient, and resilient development. Over the past few decades, International food production has experienced a remarkable expansion, with an annual growth rate of 2.6%. In 2021, global grain production reached approximately 2.8 billion tons, translating to a per capita disposable grain output of roughly 305 kg. However, despite these impressive figures, it's disheartening to note that 193 million people across 53 countries grapple with food crises, marking a 26% increase compared to 2020. This predicament is attributable, in part, to factors such as COVID-19 disruptions and extreme climate-related disasters affecting agricultural production. The outbreak and widespread impact of COVID-19 have further exacerbated global food security and nutrition concerns (3).

In our urbanizing world, technology and innovation play a pivotal role in transforming AFS. They enhance efficiency, inclusivity, resilience, and sustainability, essential for providing accessible and affordable healthy diets, thereby achieving food security and nutrition. The Research Topic highlights emerging methods for Food and Environmental Security.

Verma et al. delve into the medicinal and therapeutic properties of garlic essential oil and its use in snack foods. Garlic's rich phytocompounds confer antimicrobial, anti-inflammatory, anti-hypertensive, anticarcinogenic, antifungal, antiviral, and antioxidant attributes, enhancing both flavor and functional value.

George and Ray emphasize the importance of arbuscular mycorrhiza (AMF) in organic agriculture, promoting soil fertility and crop productivity. AMF is becoming indispensable in nature-friendly, organic agriculture, contributing to sustainable practices.

Mauricio-Sandoval et al. explore the impact of *Mangifera indica* and *Myrciaria dubia* on ice cream. These fruits enrich ice cream with phytochemicals, improving antioxidant potential and sensory appeal while maintaining health benefits.

Wang et al. investigate the factors influencing the green supply chain resilience of agricultural products, emphasizing standardization, normalization, and collaboration among stakeholders, facilitated by government policies, and subsidies.

García-Ramón et al. discusses the influence of Peruvian Andean grain flours on sliced bread. Enrichment with these grains affects dough properties, texture, color, and nutritional content, offering the potential to develop healthier, higher-quality bread.

The Research Topic entitled “*Agri-food Innovations in the quest for Food and Environmental Security”* presents a comprehensive Research Topic of original research, reviews, and systematic reviews that encompass various facets of recent advancements in the field. It is our hope that scholars will recognize the diverse nature of the challenges and be motivated to explore new avenues of study, thereby contributing to the development of Agri-food systems (AFS) that are both food and environmentally secure. Such efforts align with the Sustainable Development Goals and play a crucial role in enhancing production, nutrition, environmental sustainability, and overall quality of life. The imperative for global transformation toward more effective, inclusive, resilient, and sustainable AFS is widely acknowledged. Agricultural innovations are instrumental in achieving nutritional security on a global scale and aligning with sustainable climate objectives. All the facts and concepts have also been duly verifies by the contributing authors as Verma et al., George and Ray, Mauricio-Sandoval et al., Wang et al., and García-Ramón et al.. Collaborative efforts involving technology companies, government entities, and farmers are driving solutions within the agri-food industry. Initiatives like innovation clubs are further bolstering the sustainability, resilience, and efficiency of AFS. Recognizing the need for accelerated and redirected transformation, we envision several scenarios for the future of our world's food system. The least desirable path would involve the continuation of practices detrimental to our farms and water resources. Conversely, we can embark on a transformative journey by embracing sustainable practices that benefit both us and our planet. To achieve this, it has been crucial to acknowledge the shortcomings and inefficiencies of the current food system. Our primary objectives should encompass alleviating food insecurity and crises, enhancing agricultural practices, reducing food loss and waste, and taking significant steps toward mitigating climate change. In doing so, we can work toward constructing a new, improved global food system capable of nourishing every hungry individual worldwide.

## Author contributions

MT: Conceptualization, Formal analysis, Project administration, Validation, Writing—original draft, Writing—review & editing. MP: Data curation, Supervision, Writing—review & editing. HH: Validation, Visualization, Writing—original draft.
